# The (dis)continuing of antithrombotic drugs and its implications for occurrence of adverse cardiovascular and bleeding events in cancer patients during end of life

**DOI:** 10.23889/ijpds.v9i5.2826

**Published:** 2024-09-18

**Authors:** Sarah J Aldridge, Ashley Akbari, Adrian Edwards, Kate Lifford, Denise Abbel, Suzanne Cannegieter, Jamilla Goedegebuur, Eva Kempers, Anne Gulbech Ording, Marieke Kruip, Johanneke Portielje, Mette Søgaard, Chantal Visser, Simon Noble

**Affiliations:** 1 Swansea University; 2 Cardiff University; 3 Leiden University; 4 Erasmus University Medical Centre Rotterdam; 5 Aalborg University & Aalborg University Hospital

## Objective

For patients receiving end-of-life care, adjusting polypharmacy and deprescription of potentially harmful medicines are important impactors on quality-of-life. Antithrombotic therapies (ATT) are used by 30-50% of patients with cancer, rising to 80% in older cancer patients, and are often continued until a quality-of-life-impacting bleeding event, or a patient is physically unable to take oral medication. We aimed to describe the use of ATT and occurrence of clinical outcomes in patients with cancer during end-of-life care.

## Approach

Linked health and administrative data in SAIL were used to identify end-of-life cancer patients diagnosed in Wales, focusing on cancer types with an estimated 1-year survival time. Survival analysis was used to describe current ATT usage and to investigate the associations between ATT usage and major cardiovascular and bleeding events.

## Results

21,880 individuals were diagnosed with the pre-defined cancer types between January 2013 and December 2019, 5,660 (26%) of these were taking ATT at diagnosis. During the study period 1670 (30%) were discontinued. Median survival time of patients taking ATT at diagnosis was 169 (IQR 158-180) days, while median time to deprescription was 206 (IQR 190-224) days. Among ATT-users diagnosis, 460 (8%) and 900 (16%) experienced a major bleed or a cardiovascular event, respectively, within 1 year following diagnosis, compared to 1100 (7%) and 1930 (12%) among those who were not prescribed ATT at diagnosis.

## Conclusions and Implications

Our research highlights the need for careful assessment and management of ATT in the interest of improving quality-of-life for patients with cancer during end-of-life care.

